# The Influence of Equipment and Environment on Children and Young Adults Learning Aquatic Skills

**DOI:** 10.3389/fpsyg.2021.733489

**Published:** 2021-10-08

**Authors:** Tina van Duijn, Jonathan Leo Ng, Carolina Burnay, Neil Anderson, Luiz Uehara, Kane Cocker, Chris Button

**Affiliations:** School of Physical Education, Sport and Exercise Sciences, University of Otago, Dunedin, New Zealand

**Keywords:** children, constraints, swimming, teaching, water safety, open water

## Abstract

Learning aquatic skills is an important component of developing physical literacy in children. Aquatic skills such as floating, swimming and safe entry/exit promote engagement in different water environments and may help preserve lives in an emergency. This scoping review was conducted to evaluate the influence of task constraints (i.e., equipment) and environmental constraints (i.e., physical and social) on how children learn foundational aquatic skills. In developed countries, children are typically taught in swimming pools under direct supervision. It is also not uncommon to see children and infants learning to swim with assistive equipment (e.g., buoyancy aids). However, perhaps surprisingly, the evidence on how and where children learn aquatic skills does not uniformly promote such practices. For example, the use of flotation devices has not been proven to aid skill learning. Some researchers have advocated that children should learn aquatic skills whilst wearing outdoor clothing. One benefit of children wearing clothing is an increased capacity to practice in colder water (such as the ocean, rivers, or lakes). Overall, whilst practitioners often use equipment for various reasons it seems that not all equipment is equally useful in promoting the acquisition of aquatic skills. In less developed countries, with limited access to swimming pools and fewer resources for private instruction, a range of different open water aquatic environments and practices, such as swimming in temporarily flooded areas, have been reported. Such strategies are in urgent demand of further research given that drowning rates in less developed countries around the world exceed those in developed nations. It can be argued that learning in pools does not afford the opportunities to develop the whole range of adaptive skills that may be required in different open water environments such as navigating currents and waves, floating whilst clothed, or making life-saving decisions. Consequently, a shift toward teaching in open water environments has occurred in several countries. This review provides an evidence-base upon which practitioners can design more effective aquatic education programs for children.

## Introduction

The education of aquatic skills amongst children and young people is of fundamental importance (Brenner et al., 2003). However, many children do not receive formal aquatic skills education, and for those that do, the emphasis of instruction is typically on the reproduction of classical swimming strokes rather than the range of skills needed to recreate safely in water (Guignard et al., 2020; Willcox-Pidgeon et al., 2021). Foundational aquatic skills, such as buoyancy control (floating), treading water, re-orienting oneself, breath control, and propulsion above and below the water surface maybe essential to survive in the water (Asher et al., 1995; Stallman et al., 2008; Hulteen et al., 2018). Consequently, the aquatic skills competency of young people across the globe is likely to be variable and typically quite low. Drowning is among the 10 leading causes of death for 5–14-year-olds worldwide but in many countries, drowning is the leading cause of childhood mortality (World Health Organization., 2017). Children from developing countries are at an increased risk of drowning globally, especially those living in island countries prevalent in regions such as the Pacific Islands and South-East Asia (World Health Organization., 2021). Indeed “over 90% of deaths occur in low- and middle-income countries, with Africa recording the world's highest drowning rates and Asia carrying the highest burden of drowning deaths by number” (United Nations General Assembly., 2021: pg. 2).

The variability in aquatic skill competency amongst young people is perhaps to be expected given that the approaches to aquatic skills education globally are also quite varied. Factors that influence the way aquatic skills are taught and learnt include the physical environment and resources available, access to swimming pools or open water, availability of assistive equipment, as well as cultural and historical practices and traditions (Irwin et al., 2019). For example, it is not uncommon to see children and infants in developed countries learning to swim in heated swimming pools with direct instruction and assistive equipment (e.g., buoyancy aids, goggles, flippers, etc.). However, as this article will reveal, the evidence-base to underpin such practices is surprisingly scarce and often seems conflicting. In less developed countries, with limited access to swimming pools and fewer resources for private instruction, a range of different open water environments and aquatic practices, such as swimming in temporarily flooded areas, have been reported (Rahman et al., 2009). Such practices are in urgent demand of further investigation. As well as the physical environment, the social environment to support skill learning is important, such as for example local traditions, culture and attitudes of a community. In some communities, children learn from their family members or by being thrown in the water (de Oliveira and Peralta, 2020). In stark contrast, in many developed countries there is a long-standing tradition of learning to swim classical strokes under the close supervision of a trained teacher (Brenner et al., 2006).

This scoping review sets out to demonstrate how aquatic skills development can be influenced by equipment and environmental constraints. Aquatic practitioners such as swimming teachers and lifeguards are aware that these factors are influential on learning and routinely use and manipulate them to design education programmes (Brackley et al., 2020). However, despite this fact, we argue that neither equipment nor environment have received sufficient attention in the research literature. We chose not to undertake a comprehensive systematic review as our initial scan of electronic databases (e.g., Google Scholar, Sports Discus, and Medline) revealed only a relatively small body of peer-reviewed, empirical research studies concerning equipment and environment (28 sources: see Table 1). Therefore, the main body of this article summarizes and critically appraises this collection of work before we go on to describe some practical implications and directions for future work. Where possible we have highlighted links to theoretical frameworks in skill acquisition but this was not our primary goal. Instead, our aim was to critically evaluate the literature related to equipment and environment on how children and young people learn aquatic skills.

**Table 1:** Overview of reviewed empirical research studies (*N* = 28).

**References**	**Title**	**Research aim and design**	**Sample**	**Comparisons/intervention length and frequency**	**Post-and retention tests results**
Araiza-Alba et al. (2021)	The potential of 360° virtual reality videos to teach water-safety skills to children	To investigate the potential of Virtual Reality (VR) 360° videos as a tool to teach children about water-safety skills. Knowledge, interest and enjoyment were assessed after one of three different training methods. Randomized between-group design, measures post-intervention. No control group. One- and 8 week delayed retention tests.	*n* = 182. Aged 10–12 years.	Three instructional mediums: 360° VR videos [EXP1], traditional video [EXP2], or poster [EXP3]. The training was designed to address specific themes that contributed to drowning in coastal environments, allowing children to learn about dangers of rip currents in a relatively safe environment.	No difference was found in the learning outcomes obtained across the three mediums; however, participants in the 360° VR medium reported higher levels of interest and enjoyment.
Asher et al. (1995)	Water safety training as a potential means of reducing risk of young children's drowning.	To determine the effects of training in swimming and water safety on young preschool children's ability to recover safely from a simulated episode of falling into a swimming pool. Randomized trial with four repeated measures. Included a 12-week delayed retention test.	*n* = 109 (59 male, 50 female). Age = 24-42 months (mean 34.2 months).	Swimming ability and three basic water safety skills: deck behavior, water recovery, jump, and swim.8 or 12-week duration of water safety and swimming lessons. Biweekly lessons, groups of six children.	Both 8- and 12-week groups significantly improved swimming ability (*p* < 0.0001) but groups not significantly different from each other. Improvements in water recovery and jump and swim by end of each intervention (*p* < 0.03) but groups not significantly different from each other.
Barwood et al. (2011)	“Float First:” Trapped air between clothing layers significantly improves buoyancy on water after immersion	Exploratory study to examine the capability of children to float with freeboard (i.e., with their airways clear of the water) with (study 2) and without (study 1) different types of clothing. Order of conditions was randomized.	Study 1: *n* = 24 adults (not reviewed). Study 2: *n* = 29 children, (16 males, 13 females). Age: 12 (SD = 3) years.	Measures taken at a single time point. Measures: Buoyancy (Newtons; N), Freeboard (% of occasions achieved) upon immersion and after a 25 m submaximal swim.	Irrespective of clothing condition, experiment condition, and gender, the participants floated (i.e., the airway remained clear of the water) on 94 (± 21) % of occasions just following entry to the water and 77 (± 30)% of occasions at the end of the experiment. Children more buoyant than adults, 95± 17% freeboard.
Bunker et al. (1976)	Video-taped feedback and children's learning to flutter kick.	To investigate the effect of video-taped feedback on the learning of a continuous motor task (the flutter kick in swimming), in two groups of children. Randomized between-group (video vs. audio) design with repeated measures (Pre, post). No delayed retention test.	*n* = 36 (2 groups). Age = 4.5–6.4 years (Group 1) 6.5–8.5 years (Group 2).	Comparison for flutter kick learning using: (1) Video-tape Feedback [EXP] and (2) Auditory Feedback [CON]	No significant difference in EXP and CON for Group 1. EXP > CON for Group 2 (*F* = 4.65, *p* < 0.05).
Button et al. (2020a)	Teaching foundational aquatic skills to children in open water environments	To assess the effectiveness of teaching children water safety knowledge and skills in open water environments (i.e., harbor, river, and surf). The aquatic knowledge and skills of children were tested in a swimming pool before, immediately after, and 3 months after receiving a 3-day intensive education program. Randomized trial with three repeated measures. Included a 12-week delayed retention test.	*n* = 98. Age = 7–11 years.	3 consecutive days. Pre and post-test comparisons for aquatic skills(1) knowledge, (2) safe entry, exit and buoyancy, (3) submersion, (4) obstacles course, (5) stimulated rescue, and (6) propulsion. RT duration: 3 months after intervention.	Significant increase from pre to post-test and post to retention (*p* < 0.05) for all except submersion.
Chusaini et al. (2020)	50 m free style swimming stroke speed improvement by using hand paddle swim and parachute swim	The aim of the study was to investigate the effects of hand paddle swim and parachute swim training on increasing arm power and swimming speed. Research compared the use of a hand paddle and a parachute in a swim training program. Controlled between-group, repeated measures design. No delayed retention test. Group allocation strategy unclear.	*n* = 30. Age = 11–14 years.	8 weeks. 90–120 min × 4 sessions per week. Swimming stroke speed comparisons for swimming training using: (1) Hand paddle swim [EXP1], (2) Parachute swim [EXP2], and (3) No treatment [CON].	EXP1 and EXP2 > CON (*p* < 0.05) for mean group 50 m swim times (36.1 s [EXP1], 36.7 s [EXP2], and 37.7 s [CON]).
Clawson (1999)	The effects of toys, prompts, and flotation devices on the learning of water orientation skills for pre-schoolers with or without developmental delays	To explore the effects of toys, prompts, and flotation devices on learning water orientation skills for pre-schoolers. Pre- and post-tests were conducted using the Water Orientation Skills Checklist—Advanced (WOC-A).Controlled between-group design with repeated measures. No delayed retention test. Group allocation strategy unclear.	*n* = 42. EXP: *n* = 23. CON: *n* = 19. Age = 3–5 years.	4 weeks. 30 min × 2 sessions per week. Comparisons of water orientation skills when taught using: (1) Toys, prompts, and floatation devices [EXP] and (2) Demonstration and practice [CON].	No significant difference in water orientation skills between EXP and CON (*z*-score = 0.33, *p* < 0.05).
Costa et al. (2012)	Deep and shallow water effects on developing pre-schoolers' aquatic skills	The study focused on the process of aquatic skills achievement in two different contexts—deep and shallow water swimming pools. The aim of the study were to (i) analyse the differences between teaching methods in deep and shallow water swimming lessons for 4–5 year-old children; (ii) determine deep and shallow water differences on developing pre-schooler's aquatic skills, after 6, 12, and 18 months of practice. Independent, between-group design. No delayed retention test. Groups based on convenience.	*n* = 98 EXP1: *n* = 48 EXP2: *n* = 50. Mean age = 4.39 ± 0.49.	Comparison between teaching in: (1) Shallow water (0.6–1.0 m) [EXP1] and (2) Deep water (1.0–2.0 m) [EXP2]	EXP1 > EXP2 lessons impose water competence particularly after 6 months of practice (*p* < 0.001).Position in gliding and leg displacements were the main predictors in group differences. No significant difference for teaching methodology adopted in EXP1 and EXP2.
Croft et al. (2013)	Responses to sudden cold-water immersion in inexperienced swimmers following training.	To examine the effects of cold water training on cold shock response in adults. The study extends previous findings by considering the influence of physical activity to maintain buoyancy and subsequent swimming performance. Mixed model, independent groups design with repeated measures (i.e., pre- and post-test). No delayed retention test. Groups assigned by convenience.	*n* = 12 EXP: 3 males, 3 females CON: 4 males, 2 females. Mean age = 22.8 years [EXP], 21.8 years [CON].	1 week. Comparisons of cold shock response between: (1) Cold water immersions and mental skills training [EXP] and (2) Temperate water immersions [CON].	EXP < CON for cold shock response. EXP > CON for reduction in maximum respiratory frequency (*p* < 0.01). No significant difference for swimming duration or distance between EXP and CON.
Hue et al. (2003)	The effect of wet suit use by triathletes: an analysis of the different phases of arm movement	To examine the swimming technique of triathletes with and without wet suits. Stroke phases and arm and leg coordination during front crawl swimming were analyzed with and without a wet suit. Within-group repeated measures design. Measures taken at a single point in time (i.e., no post- or retention test).	*n* = 12 (males) Age = Not specified	Comparison of the swim velocities and arm stroke: (1) with wetsuit [EXP] and (2) without wetsuit [CON].	No significant differences in leg movements, at all velocities. EXP > CON for stroke length (+3.46% and +3.10% at paces for 100 and 50 m, respectively; *p* < 0.01); stroke index (+5.18%, +5.21%, and +5.91% at pace of 800, 100, and 50 m, respectively; *p* < 0.01); entry and catch phase (+9.81%; *p* < 0.05).
Kjendlie (2009a)	Swimming abilities are not enhanced by using a flotation suit for advanced beginners in deep water swimming teaching.	To investigate the effect of wearing a flotation suit on swimming, arm-stroking, and leg kicking performance of children in a learn-to-swim program. Children's skills were observed through video recordings, using a modified aquatic readiness assessment test. Randomized between groups design. All measures taken at post-test. No delayed retention test.	*n* = 99. Mean age = 7.4 ± 1.1 years [EXP], 7.4 ± 1.1 years [CON].	10 weeks. 1 session per week. Effects on swimming, arm stroking and leg kicking. Comparisons of using: (1) a floatation suit [EXP] and (2) not using a floatation suit [CON].	CON > EXP (NS) for swimming (3.1 ± 0.9 [CON], 3.0 ± 0.6 [EXP]); arm propulsion (2.7 ± 0.5 [CON], 2.6 ± 0.5 [EXP]); arm recovery (2.3 ± 0.7 [CON], 2.2 ± 0.7 [EXP]); and leg kicking 3.5 ± 0.8 [CON], 3.4 ± 1.0 [EXP]).
Kjendlie (2009b)	No effect of using flotation suits in gliding and floating abilities of advanced beginners in swimming teaching.	To investigate the effect of wearing a flotation suit on the floating and gliding abilities of children in a learn-to-swim program. Randomized between groups design. All measures taken at post-test. No delayed retention test.	*n* = 110. Age = 7.7 years =/– 1.3. Non swimmers with some swimming sch.ool experience	10 weeks. 1 session per week. Effects on swimming, arm stroking and leg kicking. Comparisons of using: (1) a floatation suit [EXP] and (2) not using a floatation suit [CON].	EXP = CON for floating and gliding abilities. All participants better at front floating than back. No effect of gender.
Kjendlie and Mendritzki (2012)	Movement patterns in free water play after swimming lessons with flotation aids.	To investigate differences in self-chosen aquatic movements during free play between two groups of children who both have participated in beginner swimming, one group with and the other group without the use of flotation vests. It was hypothesized that children being taught using flotation vests would be less likely to surface dive, jump, and dive during free play. Randomized between groups design. All measures taken at post-test. No delayed retention test.	*n* = 24 EXP: *n* = 11 (6 males, 5 females) CON: *n* = 13 (10 males, 3 females). Age = 6–8 years.	Free-water play behavior after 10 swimming lessons. Comparison between groups taught: (1) With floatation vest [EXP] and (2) Without floatation vest [CON].	EXP < CON for surface dives performed during free play (*p* < 0.05).
Kretschmann (2017)	Employing tablet technology for video feedback in physical education swimming class.	To determine the impact of a technology enhanced teaching scenario in PE featuring video feedback on swimming performance, particularly using a tablet computer. 25 m front crawl swim times were recorded before and after intervention. Students also interviewed about the teaching approach used. Mixed model, independent randomized groups design with one repeated measure (i.e., pre- and post-intervention).	*n* = 16 (EXP), *n* = 15 (CON). 5th grade (11 years old) PE swimming classes.	7 weeksEXP: a standardized video analysis and feedback program using a tablet computer by a trained PE teacher. CON: No additional media and technology and traditional teaching methods such as verbal feedback only.	EXP group swam faster 25 m front crawl times (*p* < 0.05), CON did not significantly improve. Students judged the video feedback scenario using a tablet computer being helpful for their learning process of improving their front crawl technique and eventually their race results.
Lao et al. (2016)	Learning to swim using video modeling and video feedback within a self-management program.	To investigate the effectiveness of assisting an adult to develop and practice swimming skills. The participant was a non-swimmer who had previously attempted unsuccessfully to learn to swim on previous occasions. A single subject design with baseline, intervention, and 12-month post-intervention phase were conducted. Single-subject design with baseline, intervention, and 12-month post-intervention phase.	*n* = 1. Age = 36 years.	13 weeks. INT: A self-management programme combined video modeling.	Improvement following intervention.
Lidström and Svanberg (2019)	Ancient buoyancy devices in Sweden: floats made of reed, club-rush, inflated skins and animal bladders.	The study presents a discussion on the material culture of traditional physical education from an ethnobiological point of view. It is argued that child-related practices connected with the bio-cultural domain and arising out of human-biota interaction have noticeably transcended time and societal changes. Qualitative ethnography.	Not applicable.	Not applicable.	Not applicable.
McCatty (1968)	Effects of the use of a flotation device in teaching non-swimmers.	To examine (a) whether the use of a flotation device accelerates the learning process of non-swimmers, and (b) whether the rate of the learning process of non-swimmers differs with different instructors, regardless of the method used. Stratified randomized within-between design with repeated measures (pre- and post). No delayed retention test.	*n* = 68 (males) Age = Not specified	14 weeks. Comparison across four groups: (1) with floatation device [EXP] and instructor A, (2) without floatation device [CON] and instructor A, (3) with floatation device [EXP] and instructor B, and (4) without floatation device [CON] and instructor B	No difference in watermanship test between EXP and CON.
Moran (2014)	Can you swim in clothes? An exploratory investigation of the effect of clothing on water competency.	To investigate the effect of clothing on water survival competencies such as swimming and floating. Swimming speed, endurance, and floating with/without clothing were explored. Physical education students with known water proficiency completed a 25 m sprint swim, a 5-min swim, and a 5-min float in swimwear and then repeated these tests a week later in clothing. Exploratory study. Within-subjects design, order of conditions not randomized.	*n* = 12 (6 males, 6 females. Age = 20–25 years.	Comparison of swimming speed, endurance and floating: (1) with clothing [EXP] and (2) with swimwear [CON].	EXP > CON for mean group swim times in 25 m sprint (27.82 ± 4.91 s [EXP], 18.51 ± 2.26 [CON]; *p* < 0.05) EXP < CON for mean group distance for 5 min swim (154.83 ± 27.57 m [EXP], 216.50 ± 47.64 m [CON]; *p* < 0.05).
Moran (2015)	Can you swim in clothes? Reflections on the perception and reality of the effect of clothing on water competency.	To investigate the effects of an aquatics education program that included the wearing of clothes in simulated water survival activities. Using a modified version of Borg's Rating of Perceived Exertion (RPE), participants were asked to estimate their exertion levels prior to and after performing a range of clothing related water activities including a 50 m sprint, a 5 min survival swim, a 15 m underwater swim, and a 5 min survival float. Paired, repeated measures (test–retest) experimental design.	*n* = 37 (21 male, 16 female). Age = 20–25 years.	Comparison to complete a range of water activities: (1) with clothing, (2) without clothing, and (3) wearing a personal floatation device.	Significant increase from pre to post-test across conditions for speed swim (50 m), endurance swim (5 min), floatation (5 min), and underwater swim (15 M).
Neufeld and Neufeld (1972)	Use of video-tape feedback in swimming instruction with emotionally disturbed children.	To investigate the effectiveness of video-tape feedback in swimming instruction for children classified as “mildly emotionally disturbed.” Observation vs. no observation of one's own performance was factorially combined with observation vs. no observation of a video-taped adept model. Independent, between-group design. No delayed retention test. Randomization unclear.	*n* = 32. Age = Not specified.	Comparison of instructional approaches: (1) Feedback on self-performance [EXP1], (2) Self-plus-model performance, (3) only model demonstration (EXP3), and (4) Conventional-instruction, no video-tape [CON]	Significant main effects for instructional approaches (*F* = 4.63, df = 1/28, *p* < 0.05). Simple effects of faster acquisition with EXP1 over EXP2 and CON (*p* < 0.10).
Parker et al. (1999)	Learning to swim using buoyancy aides.	To investigate the progress of 7-year olds learning to swim with and without buoyancy and propulsion aides. Ability-matched, within- between-group design with repeated measures. No delayed retention test.	*n* = 19. Age = 6–7 years.	2 weeks. 40 min × 5 sessions per week. Comparisons of swimming skills when learning using: (1) Buoyancy aides [EXP] and (2) Self-supported [CON].	No significant difference in swimming skills between EXP and CON.
Parsons and Day (1986)	Do wet suits affect swimming speed?	To examine if wet suits increased swimming speed. Swimmers volunteered to undertake two 30 min swims, one with and one without a wet suit. Randomized crossover trial, no post- or retention tests.	*n* = 16 (14 males, 2 females). Age = Not specified.	Comparison in 30 min swim time: (1) with wet suit and (2) without wet suit.	EXP > CON for swimming speed (~7%). EXP > CON for swimming distance in 30 min (24.9 lengths × 66 m [EXP], 23.2 lengths × 66 m [CON], 95% CI: 0.8–2.6 lengths, *p* < 0.001).
Pharr et al. (2014)	Parental factors that influence swimming in children and adolescents.	To examine the parent/child relationships concerning swimming more deeply and to determine what parental factors influence the number of days that children swim. Cross-sectional survey design.	*n* = 1,909. Collected from YMCAs in six US cities. Age = 4–17 years old (4–11 years old assisted by a parent).	Parental factors examined included: the number of days a parent swims, a parent's self-reported swimming skill level, family income, parent's educational attainment, parental encouragement to swim, frequency of a parent swimming with a child, and a parent's fear of drowning.	Children were found to swim significantly more if their parents encouraged them to swim, members of the family knew how to swim and swam with them, or their parents were not afraid of the children drowning or afraid of drowning themselves. The number of times that parents swam was the strongest predictor of the number of times children swam explaining 41% of the variance.
Rocha et al. (2018)	The acquisition of aquatic skills in preschool children: deep vs. shallow water swimming lessons.	To analyse the differences on developing pre-schoolers' aquatic skills between deep and shallow water aquatic programs after 6 months of practice. It was hypothesized that the shallow water program (while applying a controlled methodological approach) would induce an acquisition of basic aquatic skills at a higher level of proficiency. Within-between group design (not randomized), tests after 6, 12, and 18 months of practice (though no delayed retention phase without practice).	*n* = 21. Mean age = 4.7 ± 0.5 years.	6 months. Comparison for aquatic program conducted in: (1) Shallow water [EXP1] and (2) Deep water [EXP2].	No difference in aquatic readiness between EXP1 and EXP2.
Ross et al. (2014)	The development of swimming skills for African American youth: Parent and caregiver perceptions of barrier and motivation.	To investigate parent and caregiver perceptions of barrier and motivation to the development of swimming skills in African American youths. Two focus groups were conducted with parents and caregivers of swimming and non-swimming children at YMCAs in six American cities. Focus group, qualitative study.	*n* = 72 parents and caregivers of swimming and non-swimming children (12 males, 53 females, and 7 not reported). Age = Not specified.	Not applicable.	Main and sub-themes: (1) Swimming Access (Facilities; Transportation; Finances; Time) and (2) Cultural constraints to swimming aptitude (Hair; peer pressure and overconfidence; a legacy of fear; Fear and finances).
Salem (2016)	The effect of using stimulus-supported flotation tools on the learning of some basic swimming skills for blind children.	To identify the impact of using stimulus-supported flotation tools on the learning of some basic swimming skills (getting used to the water—breathing—horizontal float on the front and recovery—horizontal float on the back and recovery—breaststroke—backstroke) for blind children (B1) classification. Within-subject design with repeated measures (pre- and post-test).	*N* = 10. Age and gender = Not specified.	8 weeks. 3 × 1 h modules per week. Compared six basic swimming skills before and after education intervention.	Significant improvements (*p* < 0.05) by post-test in all six basic swimming skills.
Scurati et al. (2019)	Toward a Safe Aquatic Literacy: teaching the breaststroke swimming with mobile devices' support. A preliminary study	To compare the effects of a short-delayed video-feedback provided by a mobile device during a breaststroke teaching program compared to the standard teaching approach which includes teachers providing conventional feedbacks, comments and corrections, only by means of gesture and verbal communication. Gender-matched between-within groups design (pre, post). No delayed retention test.	*n* = 16 (8 males, 8 females). Mean age = 20.6 ± 0.5 years.	8 weeks. 1 × 45 min session per week. Comparison for breaststroke learn-to-swim program with: (1) video feedbacks by mobile devices [EXP] and (2) conventional feedbacks, comments and corrections [CON].	No significant interactions between EXP and CON (Mixed-model ANOVA).
Willcox-Pidgeon et al. (2021)	Exploring children's participation in commercial swimming lessons through the social determinants of health.	To investigate the impact of two social determinants of health, socio-economic status and geographical remoteness of residential location on participation in swimming and water safety programs. It also provided a situational analysis of the swimming and water safety skills that are taught in commercial swimming lessons and analyzed children's skills and achievement levels. Cross-sectional retrospective study.	*n* = 43,201 records (50.8% males; 53.1% aged 5–7 years) Age = 5–12 years (Median Age; 7.0 ± 1.98 years).	Not applicable.	Socio-economic status (SES) had a significant effect on achieving the minimum benchmark skill of 50 m freestyle (13.6% [Low SES] vs. 26.1% [high SES]) (χ^2^ = 81.78; *p* < 0.05). Most common skill taught by swim schools were freestyle (87%) and backstroke (83%). 21.3% of schools did not teach water safety skills. 9.2% of children were taught rescue skills. 40% of 12-year-old children did not achieve the National Benchmark of 50 m freestyle.

Based on Arksey and O'Malley's (2005) framework, the process of this scoping review started by identifying the research question (stage 1), after which the research team identified potentially matching studies and collected them in a common file (stage 2). In conducting this initial scan, we used combinations of relevant search terms such as “aquatic skills, children, education, skill acquisition, swimming, teaching, water safety, and young people.” Our literature scan utilized comprehensive electronic databases that were deemed most likely to cover publications concerning aquatic skill acquisition. We also consulted reference lists, relevant organizations (i.e., Water Safety New Zealand, World Health Organization) and hand searched key journals (i.e., International Journal of Aquatic Research Education). During stage 3, all relevant studies (*n* = 28) were identified and selected. Selection criteria included scientific rigor, peer-review, availability in the English language. Studies were only selected if children or young people's learning of aquatic skills or competency was directly investigated, and if at least one of the constraints of interest (i.e., equipment, environment, socio-cultural context) was investigated. The studies were charted (stage 4) and described based on a selection of factors (i.e., age of participants, presence of control group, main research question, main findings, relevance for topics). During the final stage, relevant information from the selected studies was summarized, collated, and reported. Members of the writing team were allocated responsibility for lead writing different sections depending on their interests and experience. Once a complete draft of the scoping review had been finished all co-authors read it and suggested edits for the lead author to complete submission.

## The Use of Equipment in Education of Aquatic Skills

In this section, we discuss research on the use of equipment in teaching of aquatic skills. The section is further sub-divided into common types of equipment used, namely: assistive devices, toys, clothing and emerging technology.

### Assistive Devices

**Figure 1:**
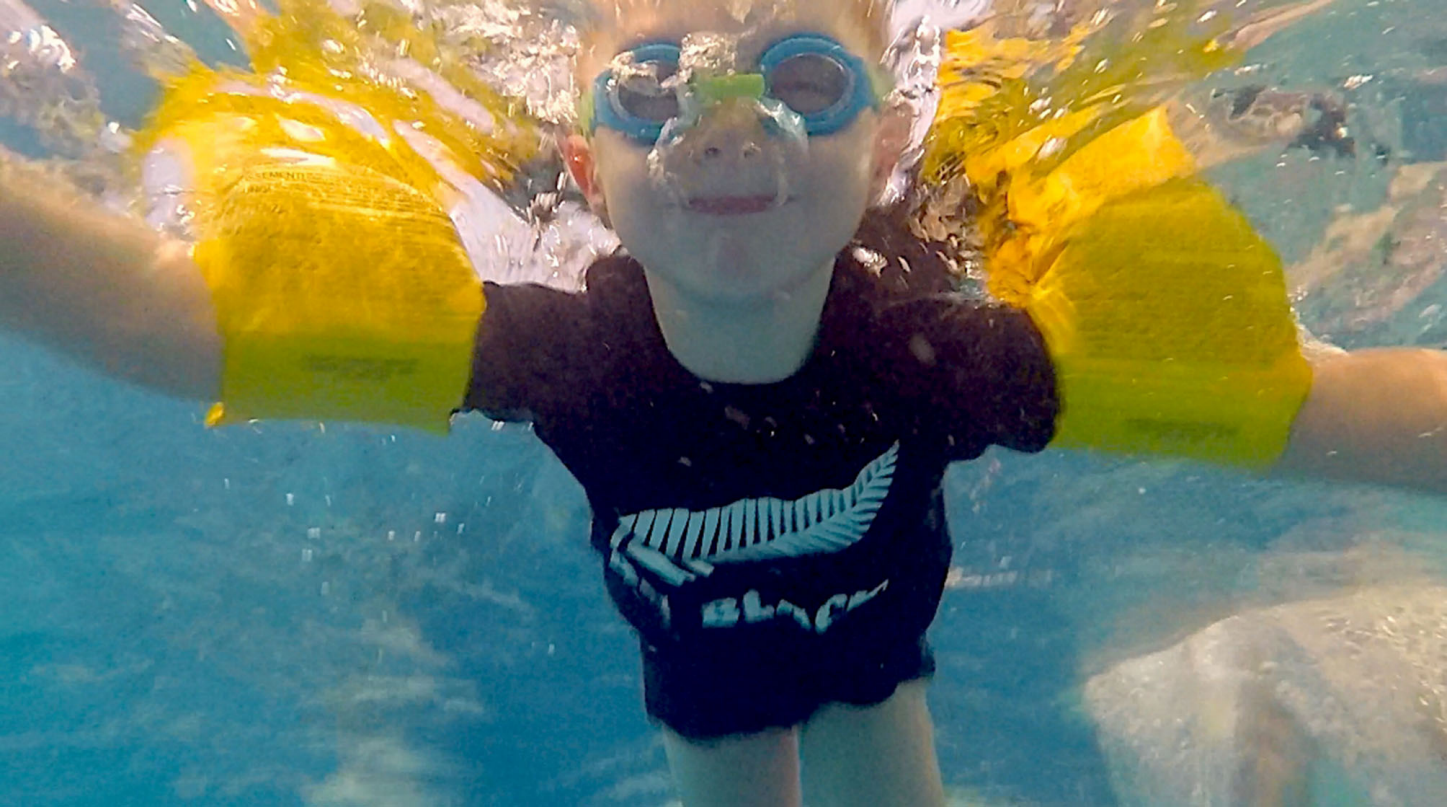
A child wearing water wings while swimming.

Assistive equipment such as kickboards, flippers, water wings (or armbands: see Figure 1), hand paddles, pool buoys and lifejackets (or personal flotation devices) are used to aid aquatic skills performance and learning (e.g., Chusaini et al., 2020). The use of flotation aides, in particular, is controversial (Langendorfer, 1987). For example, some practitioners might defend the use of flotation aides as a safety precaution when teaching large groups of learners. Another argument in favor of using floating equipment is that children are often more engaged and confident with such devices and that they may provide an important boost in confidence (Parker et al., 1999). Such views are consistent with the Challenge point framework of motor learning (Guadagnoli and Lee, 2004) which suggests that the difficulty of a practice activity should be adjusted to the level of the learner *via* equipment or informational prompts. However, some critics have condemned floating devices as unnecessary and propose that they may inhibit the learning of independent swimming or, worse still, provide a false sense of security that may lead to risky behavior (e.g., Kjendlie and Mendritzki, 2012; Quan et al., 2018). Another criticism of flotation devices attached to the child's body is the resulting alteration in the center of buoyancy that places the child in an unnatural body position (Langendorfer, 1987).

The first recorded use of floating devices attached to a body to teach swimming dates back at least 3,000 years ago. Historic illustrations depict the use of inflated animal skins and bladders, presumably of goats, by the Assyrians as swimming floats (Lidström and Svanberg, 2019). Although the use of assistive equipment is common in contemporary education programs (e.g., see Figure 1), there is a surprising dearth of evidence to support their use. Kjendlie conducted experimental studies where ~100 children, aged 7, were taught to swim with or without a floating vest (Kjendlie, 2009a) or with floating suits (Kjendlie, 2009b). The author found no statistical differences for floating or gliding abilities (Kjendlie, 2009a) nor for swimming, arm stroking and leg kicking performance (Kjendlie, 2009b) when the children used the floating devices. Similarly, Kjendlie and Mendritzki (2012) analyzed the effect of using floating vests in teaching 6–8-year-olds surface diving, jumping, and diving during free play. Although the results showed no significant differences for breathing, diving, and water entry skills, the group wearing the flotation aid had significantly fewer surface dives and seemed to be less likely to perform vertically-oriented movements compared to the control group. The authors concluded that the use of flotation aids can make children somewhat less competent in a range of different aquatic skills (Kjendlie and Mendritzki, 2012). Parker et al. (1999) found that learning to swim either with multiple flotation and assistive devices (i.e., kickboards, polystyrene bubbles, flippers) or with just one assistive device (i.e., kickboards) was equally effective for 7-year-old children. These authors conclude there was an absence of transfer of performance quality from aides-based practice to unaided swimming. Another study analyzed the impact of the use of kickboards and pool buoys in improving swimming skills of secondary school students (Ramón and Valero, 2018). The unaided group improved more than the group using the aids, suggesting a negative effect of the use of propulsion aids in learning swimming skills. Salem (2016) did show a positive effect of using ‘stimulus-supported flotation tools’ for a small group of blind children. The children underwent an 8-week education programme using a range of equipment designed to improve the physical skills and confidence of the students. Significant improvements in floating, swimming and breathing control were found although notably there was no control group, nor retention and transfer tests and limited details provided about the sample and teaching programme (Salem, 2016).

Overall, there is a lack of positive evidence in favor of using assistive equipment to teach aquatic skills to children and young adults, although admittedly more research is required. For instance, there is no scientific evidence about how long or how often early learners might use assistive devices or about the best means to transition to unaided swimming. Until such research is conducted, it might be advisable to follow Langendorfer's (2012) practical recommendations: “the use of flotation devices when teaching young swimmers should fit the needs of each individual” (pg. 4). Instructors should evaluate each child individually alongside the goals of their education and decide if the use of assistive devices would be beneficial based on that individual's needs. Such a general guideline sits well with current motor learning theory recommending a learner-centered approach to practice design (Button et al., 2020b).

### Toys

Another type of equipment that is commonly used in swimming programs for young children are toys. Toys are essentially motivational aids that promote behaviors in the water that children would otherwise not exhibit. There are a wide range of aquatic toys, some of which are designed to float whereas others sink to promote underwater play. Toys can serve as a distractor from possible apprehension of the new aquatic environment (Langendorfer, 1987). However, we found only one experimental study that has specifically addressed the impact of using toys in aquatic skill acquisition. Clawson (1999) analyzed the effect of the use of toys in teaching pre-schoolers water orientation and swimming skills. The results showed that the use of toys during swimming classes had a positive influence on entering the water and getting the face wet but they did not enhance water orientation nor swimming skills. Of course, as children get older the likelihood of toys having the same motivational benefits will inevitably decrease as their appeal wanes.

### Clothing

The education of aquatic skills needs to address the factors associated with drowning incidents more directly (Button, 2016; Stallman et al., 2017). Drowning often occurs due to accidental falls into deep water when individuals are wearing typical outdoor clothing (e.g., whilst fishing or wading across a stream on a tramp). In New Zealand, for instance, accidental immersion represented 32% of all fatal drowning incidents between 2015 and 2019 (Water Safety New Zealand., 2021). However, no literature on the influence of clothing on learning water competency in children was found. One study showed that street clothing initially increases buoyancy through the trapping of air between layers of clothing (Barwood et al., 2011)^1^. The authors acknowledge that the clothing buoyancy effect is temporary as air bubbles are released over time and the added weight of clothing can become more problematic to maintain flotation. A small but interesting body of work on the effect of young adults wearing clothes while swimming has shown that clothing significantly reduces swimming speed and swim endurance (Moran, 2014) and that even competitive swimmers find some tasks (i.e., sprint swimming, underwater swimming, survival floating) harder to perform when wearing clothes (Moran, 2015). These studies suggest that exposure to swimming in clothing in water safety and swimming programs may help children to understand the extra effort required and better prepare them for unexpected immersion incidents.

Previous studies have shown that the use of wetsuits improves swimming speed (Parsons and Day, 1986) and swimming coordination (Hue et al., 2003). However, these studies were conducted in competition level triathletes. An additional benefit of children wearing clothing, such as wetsuits, is an increased capacity to practice in colder water (such as the ocean, rivers, or lakes). This would expand the range of possible environments in which children could learn (see Environment section).

### Emerging Technologies

Recently, video technology has become easily accessible due to the increased functionality and use of mobile devices such as phones and tablets. This has led to an increased use of video feedback both in competitive swimming and as a teaching tool in learn-to-swim programmes (Kretschmann, 2017). Some studies have shown a positive effect of using mobile devices to offer video feedback when young adults were learning swimming skills, such as breaststroke (Ferracioli et al., 2013; Scurati et al., 2019) and freestyle (Lao et al., 2016). Two studies dating back to the 1970's have addressed the effect of video feedback in teaching children swimming skills. A first study showed no effect of video feedback in 3–6 years old children' swimming performance (Neufeld and Neufeld, 1972). A second study showed that 6–8 years olds benefited from video feedback when learning the flutter kick, but no effect was observed in younger children (4–6 years old) (Bunker et al., 1976). A promising recent study by Araiza-Alba et al. (2021) suggests that virtual reality might become a powerful tool to help educate children about how to identify risks in open water environments such as rips at the beach (Araiza-Alba et al., 2021). There is a need for further studies investigating the use of new and increasingly accessible technologies such as waterproof cameras and virtual reality in teaching children aquatic skills.

## The Influence of the Environment on Learning of Aquatic Skills

### Physical Environment

The physical environment in which learners develop aquatic skills plays an important role. By adapting to demands of the environment, a learner develops new skills, such as water safety competencies, that are effective and fitting with their personal action capabilities. Traditionally, most learners in the developed world are taught to swim in enclosed aquatic environments such as swimming pools (Button et al., 2020b). However, it has been argued that learning in pools may not afford the opportunities for children to develop the whole range of adaptive skills that are required in different open water environments (Asher et al., 1995). Many drowning incidents happen in open-water environments (World Health Organization., 2021), therefore aquatics education programmes should consider including opportunities for learning in open-water environments. Introducing learners to various physical environments (e.g., streams, lakes, ocean, etc.) has recently been signaled as important to foster the development of robust and adaptable aquatic skills (Guignard et al., 2020). However, there is a lack of research into the effects of teaching children aquatic skills in dynamic, open water environments where a range of factors such as currents, temperature and surface waves have the potential to influence skills learned. Importantly, Kjendlie et al. (2013) suggest it cannot be assumed that learners would be able to effectively transfer the aquatic skills from a closed, predictable environment of swimming pools into the dynamic open water environment.

**Figure 2:**
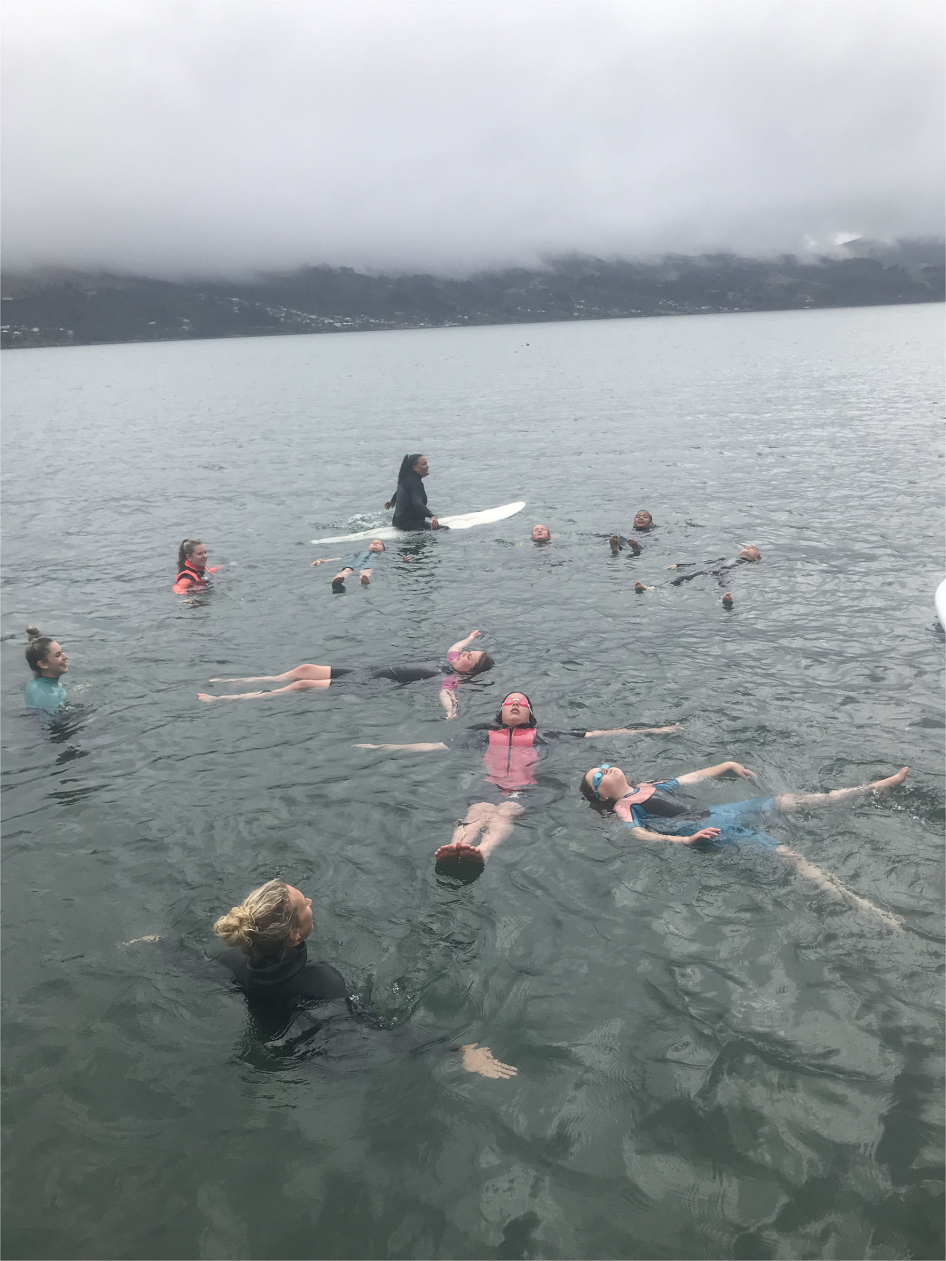
Children learning how to float in an outdoor environment, under close supervision.

Recent research by Button et al. (2020a) appears to be the first investigation into the efficacy of aquatic skills education in an open water setting. The authors had 98 children (7–11 years old) undergo a water safety education programme that included supervised practice sessions at open water harbor, river and ocean environments (see Figure 2). Despite the brief duration of the education programme (3 days) the results demonstrated that most children significantly improved their aquatic competencies (i.e., both physical skills and safety knowledge) and retained them up to 3 months later. Button et al. (2020a) contrasted their data with children of the same age taught a similar programme in swimming pools and noticed that whilst similar levels of skill improvement were obtained, the retention of skills was superior amongst children taught in open water environments. Ongoing research by this group is investigating how transferable skill learning is between different aquatic environments.

Studies investigating the impact of swimming lessons at different depths of water have shown mostly benefits of exposing learners to both deep and shallow water (Costa et al., 2012; Rocha et al., 2018). Costa et al. (2012) explored whether 4-year-olds who had swimming lessons in deep water developed skills differently compared to children who had learned to swim in shallow water. Three groups of children were included in the study who had 6-, 12-, or 18-months experience of swimming, respectively. Various skills associated with floating, propulsion, and breath control were assessed. For most of the skills (~80% of skills assessed), there was a significant benefit of being taught in shallow water. However, this effect was strongest for children with 6 months of experience while benefits of shallow water training were only identified for 35% of children with 12 months experience, and none of the skills assessed in children with 18 months experience showed a benefit. Costa et al. (2012) suggested that training in a shallow-water environment might be more beneficial for novices and inexperienced learners than practicing in deep-water^2^. Supplementing the work by Costa et al. (2012), Rocha et al. (2018) investigated how skills developed in children who had no previous experience in swimming. In their study one group completed lessons in shallow water (0.7 m) and the other group completed lessons in deep water (1.3 m). Similar to Costa et al. (2012), children who practiced in shallow water developed better skills in horizontal buoyancy (i.e., floating / gliding in a dorsal and ventral body position and kicking in a ventral position without a flutter board).

Around the world, attempts are being made to integrate different physical environments in aquatic skills education but such an aim can be challenging for a range of reasons. For example, a pilot project conducted in Switzerland showed that conducting swimming lessons outdoors in the Swiss summer was feasible, yet it required adaptations to deal with cold water, unpredictable weather, anxious children, parental supervision and organizing of lessons (Amberg et al., 2020). In many countries, cold water immersion poses a considerable risk. A review by Datta and Tipton (2006) shows that either the initial “cold shock” response (inspiratory gasp, hyperventilation, hypocapnia, tachycardia and hypertension) or subsequent hypothermia are both responses that override autonomic respiratory control and may lead to drowning. Button et al. (2015) reported that immersion in cold water (10°C) affected swimmers of different skill levels similarly (i.e., physiologically and functionally). However, to our knowledge, there is no peer-reviewed literature that investigates the effects of children practicing aquatic skills in cold water (i.e., <12°C). Some promising research was conducted in New Zealand by Croft et al. (2013) who showed how repeated cold water immersions could assist young adults to cope with cold shock. They found that with training (10 × 3 min immersions in 15°C) participants were able to significantly “blunt” the cold shock response (i.e., reduce inspiratory gasp, hyperventilation, heart rate, etc.). A range of other logistical challenges exist in open water aquatic skills education such as managing risks for groups of learners and having sufficient instructors with knowledge of the environment to supervise sessions. Nevertheless, acknowledging the likely threats that climate change could pose in the future (i.e., more frequent extreme weather events, rising sea levels, flooding, etc.), it seems that practicing aquatic skills in a range of physical environments is an astute and necessary part of gaining water competency.

### Socio-Cultural Environment

As well as the physical environment, there is good reason to suspect that numerous social and cultural factors influence aquatic skill learning. For example, statistics and reports show that there are clear effects of race (Saluja et al., 2006), gender (Howland et al., 1996), age (Quan and Cummings, 2003), and socio-economic status (Leavy et al., 2016) on drowning incidences. In terms of race, African American, Native American and Latino children are 2–8 times more likely to drown compared with white children from the same countries (Olaisen et al., 2018). Regarding socio-economic status, many families who live in remote and rural areas do not have adequate resources to provide education for their children to learn how to swim. Willcox-Pidgeon et al. (2021) investigated the role that socio-economic status might have in the development of water competencies in over 43,000 Australian children. They report that children from low socio-economic areas and remote locations were less likely to be able to swim 50 m continuous freestyle. Similarly, parents of US African American children identified structural barriers such as the lack of time, money, or facilities as some of the reasons of their children not having taken swimming lessons (Ross et al., 2014).

Leavy et al. (2016) conducted a review of all drowning prevention interventions for children and young people with a focus on differences between high, low and middle income countries (Leavy et al., 2016). They found that parental supervision was one of the most effective injury prevention strategies, especially in high income countries (HICs), and they found it to be a low-cost, socially acceptable method to prevent drowning across all cultures (Wallis et al., 2015). A study by Pharr et al. (2014) found that the number of times the parents swam was the strongest predictor of the number of times their children swam themselves. Cyclical patterns in the data suggested generational trends of parents encouragement, members of the family knowing how to swim and swimming with them, and a fear of drowning related strongly to whether US children are encouraged or discouraged from swimming (Pharr et al., 2014).

Beattie et al. (2008) argue that rural water safety strategies need to include the community, family and individual level. This is best achieved through a community development framework, by working with local organizations and involving the commitment, local pride, and knowledge of community champions. As an example, a program called “Water Safety in the Bush” employed a flexible, community development model to meet the special requirements of remote and isolated communities. A water safety instruction program based on a Royal Life Saving Society of Australia curriculum was adapted to meet local priorities and strategies for sustainability (Beattie et al., 2008). The authors of the report concluded that effective health promotion requires going beyond simplistic models of delivering urban models in rural areas. Another example is a study on children in Bangladesh, which showed a substantial and statistically significant reduction in relative risk of drowning in SwimSafe children as compared to the matched non-SwimSafe children (Talab et al., 2016). There is growing recognition that water safety education programmes are most effective when contextualized to socio-cultural factors and targeted toward different ethnic groups. For example, Phillips (2020) recently proposed a water safety framework for Māori (indigenous NZ people). On average Māori account for 20–24% of all preventable and non-preventable drowning fatalities, despite comprising only 15% of New Zealand's population. The Wai Puna model includes three core concepts: whakapapa (genealogy and identity), mātauranga (Māori knowledge and ways of knowing) and tikanga (customs and practices). Phillips (2020) suggests that “Wai Puna provides the foundation for conceptualizing Māori water safety in a New Zealand context and a way forward for other indigenous communities around the world to redefine water safety and drowning prevention from their distinct worldviews that reflect their unique beliefs and attitudes to water and thus to water safety” (pg. 1). Robertson (2010) also stresses the importance of cultural diversity and inclusion in learn-to-swim programmes in the school community. In a survey of 2,553 NZ schools, she found that school leaders were aware of cultural reasons why some students might not feel comfortable swimming (e.g., due to religious beliefs and the need to dress modestly). The author also stressed the importance of providing support to students who may not have grown up around regional bodies of water, for example recent migrants. Unfortunately, language and literacy barriers as well as culturally inappropriate strategies are common in many water safety interventions conducted to date (Solomon et al., 2013).

## Discussion

Aquatics educators routinely consider equipment and environment as influential factors for learners, however to what extent does the scientific literature underpin common practice? This scoping review aimed to investigate the effects of equipment and environment on children and young people's learning of aquatic motor skills. Our literature search revealed a relatively small body of empirical studies (*N* = 28) published to date. Furthermore, several studies showed methodological limitations or limited detail that hamper us from drawing firm conclusions and implications. For example, whilst 18 studies included randomized—controlled groups, fewer had random allocation to groups (*n* = 12) and even fewer (*n* = 5) included a delayed retention test. Furthermore, the skills that were assessed differed between each study, which limits the generalizability of the results. Similarly with regards to environment, although it is increasingly recognized that physical and socio-cultural factors are influential for learning, much of the research we reviewed was descriptive and the implications for learning are yet to be elucidated. In this final section, we will highlight some of the common findings, suggest directions for future research and conclude with practical recommendations.

Whilst assistive equipment like hand paddles and fins can provide a training benefit to more advanced learners, we found very little evidence to suggest that they should be used with early learners. Two published studies that investigated the use of assistive devices in learning to swim show opposite effects, and a single study on the use of toys showed a positive effect of toys only on motivational factors during swim classes. Video feedback through tools such as waterproof mobile devices is increasingly accessible for aquatic skill education. However, this review only identified two studies that exist in children's learning of swimming skills, which show mixed results, and dates back to the 1970's. While it is clear that the energetic requirements of swimming in clothing are increased due to additional weight and drag resistance, the effect of wearing different types clothing on skill learning has not been directly tested.

Similar to the use of equipment, the effect of learning in different environments—either physical or socio-cultural—is not well-documented. Based on the present review, there may be a benefit of developing foundational aquatic skills in shallow water, but there is not enough evidence to provide a specific recommendation in this direction. Although research in an older cohort has shown a potential use for training in cold water to reduce the cold shock response, there are no studies, to our knowledge, that investigated the effects of practicing aquatic skills in cold water on children's learning of aquatic skills. Finally, there is emerging evidence that exposure to a range of aquatic environments (e.g., open water bodies of water as well as swimming pools) is beneficial for the acquisition and retention of skills. A necessary extension of this work is to ascertain whether the benefits of children learning in open water are transferable across different physical environments. The social and cultural environment is assumed to play a significant role in the learning of aquatic motor skills. Based on risk factors for drowning, it can be assumed that skill learning is affected by access to resources (materials, pools, and tuition) as well as socio-cultural factors, such as attitudes and behaviors in the surrounding social environment, cultural inclusion in schools, and aquatic behavior of the family (e.g., Leavy et al., 2016).

### Recommendations for Future Research

The field of aquatic skill learning desperately needs more evidence-based instruction tools and methods (Stallman et al., 2008). Future research should focus on randomized, controlled learning studies that include delayed retention tests, and where the instruction content, teaching style and other potential confounders are kept stable. In our opinion, a priority is to determine the effects of different physical environments on skill acquisition. Such a strategy has potential for bypassing a common concern about cost and access to swimming pools (e.g., Stevens and NZCER, 2016). For example, it will be valuable to understand how different environmental factors such as water flow speed, waves, depth, temperature, visibility, turbulence (etc.) affect the development of aquatic skills in children (Kjendlie et al., 2013). Although emerging evidence shows children's aquatic skills can be effectively learnt in a range of open water environments (Amberg et al., 2020; Button et al., 2020a), this approach has yet to be confirmed in a large scale project alongside a matched control group. We can attest from first-hand experience that testing and conducting learning studies in open water environments poses numerous challenges: safety requirements, weather dependence, temporal demand, managing group sizes, and a range of dynamic factors that are difficult to predict (i.e., temperature, depth, visibility). An alternative to conducting research in open water is to simulate aspects of it that are the focus of the investigation, for example in a flume pool or “lazy river” with adjustable current and temperature (Guignard et al., 2020). The cognitive and motor demands placed on an individual should be represented as realistically as possible to ensure external validity when these skills are assessed.

As mentioned earlier, existing research investigated a range of aquatic skills, which makes it hard to draw general conclusions. The general approach in swim teaching is still fairly traditional, i.e., coaches seek to teach a “perfect” and consistent swimming technique. However, in times when a focus on developing water safety and survival skills is becoming more and more necessary, assessment of aquatic competence must go beyond the assessment of swimming competency, but should also include elements of aquatic survival skills such as the ability to enter and exit the water safely, navigate obstacles, stay afloat, clothed swimming, and make correct decisions. Recently, Stallman et al. (2017) created a list of water safety competencies based on the most common drowning causes and suggested that these competencies or skills should be included in water safety and swimming instruction. This list could be a starting point for future studies. Attempts have been made to develop a testing battery to assess these competencies (Button et al., 2019), however, this testing battery is in need of further validation. Future research should investigate the best utility of different types of equipment, and of scaling the equipment to the action capabilities (e.g., ability, strength, height, buoyancy) of the learner. Such equipment could be positively or negatively buoyant, propelling or adding drag, hindering movement (such as regular clothing) or supporting it (such as wetsuits and lifejackets).

### Links to Motor Learning Theory

When discussing the effects of equipment and environment on learning, a link to ecological dynamics seems most obvious: equipment and environment constrain the learner's actions, create affordances that may trigger motor adaptations in a learner and to a greater or lesser extent represent the information-movement couplings that are required to become physically literate (Rudd et al., 2021). Teachers might purposely choose equipment and environment to create task and environmental constraints, in order to support exploration and to stimulate the development and adaptation of effective movement solutions (Guignard et al., 2020). Indeed, whether aware of the theory or not, aquatic practitioners such as swimming teachers and lifeguards seem to already routinely use and manipulate factors linked to equipment and environment to design education programmes (Brackley et al., 2020).

The interaction of personal, task, and environmental factors in determining learning potential is also reflected in the “Challenge point framework” (Guadagnoli and Lee, 2004). The framework posits that an optimal challenge point exists, where the potential learning is maximized (i.e., the task is difficult enough to trigger learning) but detriment to performance in practice (i.e., errors due to high difficulty) is minimized. Difficulty level needs to be at the optimal challenge point for optimal learning, which may be a very different level of difficulty for different learners. Based on the framework, the learning potential that arises from a specific task further depends on the interaction of skill level task complexity and environment, as “motor tasks represent different challenges for performers of different abilities” (Guadagnoli and Lee, 2004, p. 212). So, for example, while one learner is at their optimal challenge point and effectively learns to float with a small floating ring under their back, another learner may not be challenged enough to learn at all, and yet another may be overchallenged: they may need more support (e.g., a floating belt) in order to perform well. This argument might explain the opposite findings regarding the benefits of floating equipment, for example, that were summarized earlier in this review. As suggested frequently in this special issue it would be of interest to investigate how strategies of equipment scaling can be used effectively to facilitate a learner-centered approaches to aquatic skill education. This scoping review did not include a detailed analysis of research on the effect of individual constraints on children's learning of aquatic skills, as these constraints are typically less amenable to manipulation by practitioners. A separate review in the future should be conducted to investigate the roles of those individual factors.

## Conclusion

Given that young children are considered at a high risk of being involved in drowning incidents, emphasis should be placed on encouraging the development of aquatic skills from a young age. Whilst aquatics practitioners often use equipment for various reasons, it seems that not all equipment is proven to be useful in promoting skill acquisition in children. Thus, we hesitate to draw any firm recommendations for practice due to the lack of direct research evidence. Conversely, the environment is not widely used as a task constraint by practitioners, although the research we explored collectively points toward a benefit of providing environments with a variety of challenges and opportunities for adaptation. It has also become clear that the choice of aquatic environment may be a useful tool to target specific skills that children should develop. For example, to enable the learning of floating in turbulent water, an environment where the water is moving and inconsistent may be chosen, rather than still water. With socio-cultural constraints such as access to swimming pools or qualified instructors being potential barriers to the development of foundational aquatic skills, targeted, and contextualized education strategies seem best placed to address the global drowning problem. For example, governments might consider whether school-based, community, or family-centered interventions are most effective at reaching children of all ages and backgrounds. In addition, the inclusion of teaching skills relating to aquatic competence, beyond that of swimming, should be mandated and across world regions (Stallman et al., 2017). In general, aquatic skills should be developed with their use in mind: the more realistic and life-like a learning scenario, the better the chances of transfer and retention of aquatic skills.

## Author Contributions

TD wrote the Introduction, co-wrote the final version, and prepared the manuscript for submission with CBut. JN prepared the table and references and wrote a first draft of the discussion. NA co-wrote the section on environmental constraints with LU. CBur co-wrote the section on equipment with KC. CBut wrote the abstract. All authors conceived the idea jointly and assisted with the scoping review.

## Funding

This study was funded by the Swiss National Science Foundation (P2SKP1_187632).

## Conflict of Interest

The authors declare that the research was conducted in the absence of any commercial or financial relationships that could be construed as a potential conflict of interest.

## Publisher's Note

All claims expressed in this article are solely those of the authors and do not necessarily represent those of their affiliated organizations, or those of the publisher, the editors and the reviewers. Any product that may be evaluated in this article, or claim that may be made by its manufacturer, is not guaranteed or endorsed by the publisher.

## Footnotes

^1^However, this was not a learning intervention. Participants' acute responses to wearing clothing were assessed at a single time point.

^2^However, the authors noted that the depth of the pool may have influenced the methodologies employed by swim teachers (i.e., use of flotation armbands) and this may have had an effect to the skills that were developed by the children under their care.
